# Cytotoxic, Cytostatic and HIV-1 PR Inhibitory Activities of the Soft Coral *Litophyton arboreum*

**DOI:** 10.3390/md11124917

**Published:** 2013-12-10

**Authors:** Mona S. Ellithey, Namrita Lall, Ahmed A. Hussein, Debra Meyer

**Affiliations:** 1 Department of Biochemistry, University of Pretoria, Pretoria 0002, South Africa; E-Mail: u12000541@tuks.co.za; 2 Department of Plant Science, University of Pretoria, Pretoria 0002, South Africa; E-Mail: Namrita.Lall@up.ac.za; 3 Department of Chemistry, University of the Western Cape, Private Bag X17, Belleville 7535, South Africa; E-Mail: ahmohammed@uwc.ac.za

**Keywords:** red sea, *Litophyton arboreum*, HIV-1 protease, HIV-1 reverse transcriptase, cytotoxicity, real time cell analysis

## Abstract

Bioassay-guided fractionation using different chromatographic and spectroscopic techniques in the analysis of the Red Sea soft coral *Litophyton arboreum* led to the isolation of nine compounds; sarcophytol M (**1**), alismol (**2**), 24-methylcholesta-5,24(28)-diene-3β-ol (**3**), 10-*O*-methyl alismoxide (**4**), alismoxide (**5**), (*S*)-chimyl alcohol (**6**), 7β-acetoxy-24-methylcholesta-5-24(28)-diene-3,19-diol (**7**), erythro-*N*-dodecanoyl-docosasphinga-(4*E*,8*E*)-dienine (**8**), and 24-methylcholesta-5,24(28)-diene-3β,7β,19-triol (**9**). Some of the isolated compounds demonstrated potent cytotoxic- and/or cytostatic activity against HeLa and U937 cancer cell lines and inhibitory activity against HIV-1 protease (PR). Compound **7** was strongly cytotoxic against HeLa cells (CC_50_ 4.3 ± 0.75 µM), with selectivity index of SI 8.1, which was confirmed by real time cell electronic sensing (RT-CES). Compounds **2**, **7**, and **8** showed strong inhibitory activity against HIV-1 PR at IC_50_s of 7.20 ± 0.7, 4.85 ± 0.18, and 4.80 ± 0.92 µM respectively. *In silico* docking of most compounds presented comparable scores to that of acetyl pepstatin, a known HIV-1 PR inhibitor. Interestingly, compound **8** showed potent HIV-1 PR inhibitory activity in the absence of cytotoxicity against the cell lines used. In addition, compounds **2** and **5** demonstrated cytostatic action in HeLa cells, revealing potential use in virostatic cocktails. Taken together, data presented here suggest *Litophyton arboreum* to contain promising compounds for further investigation against the diseases mentioned.

## 1. Introduction

HIV/AIDS is one of the most devastating diseases in the world with approximately 34 million people living with the virus in 2010 and approximately 2.7 million new infections in that same year [1]. Antiretroviral therapy (ART) successfully reduces infection and decreases symptoms; but, the emergence of viral drug resistance, due to drug-induced mutations in viral genes, renders treatment ineffective. This underscores an urgent need to develop new anti-HIV drugs [2,3] with fewer side-effects to improve patient compliance.

The use of anti-HIV drugs as cancer treatments is not new. Azidothymidine was studied as an antineoplasic in the 1990s, but despite promising *in vitro* data, clinical trials showed little antitumor activity. HIV protease inhibitors were developed in the early 1990s, and their subsequent incorporation into highly active antiretroviral therapy (HAART) has profoundly changed the natural history of HIV infection. The potential antitumor properties of these drugs have been investigated because of their success in treating HIV-related Kaposi’s sarcoma. HAART’s effects on Kaposi’s sarcoma did not always correlate with immune reconstitution, and activity against other solid and haematological malignancies has been established. Inhibition of tumor-cell invasion and angiogenesis were properties first ascribed to HIV protease inhibitors; these drugs have pleiotropic antitumor effects, including inhibition of inflammatory cytokine production, proteasome activity, cell proliferation and survival, and induction of apoptosis. HIV protease inhibitors are thus a new class of anticancer drugs with multiple effects, and other anti-HIV drugs might hold similar promise [4].

Marine organisms as a source of natural products delivered numerous novel compounds with sensational multiple pharmacological properties. During the past 20 years, thousands of novel compounds and their metabolites with diverse biological activities ranging from antiviral to anticancer have been isolated from various marine sources. The use of marine natural products as anti-HIV agents has also been described [5] with a number of potential lead compounds identified. In computational science, natural products have long captured the attention of medicinal chemists due to the diversity of their chemical scaffolds, potentially lower toxicities and bioactive substructures [6].

The Red Sea still contains a large number of uninvestigated organisms (flora and fauna). Exploration of untapped regions of this unique resource for the discovery of bioactive natural compounds is an urgent task due to the impending environmental changes that can occur to the wild flora as human encroachment continues. *Litophyton arboreum* is a common octocoral, widely distributed on the Red Sea coral reefs. A previous chemical study of *L. arboreum* growing in different parts of the Red Sea showed the presence of cembranoide diterpenes [7], which had moderate cytotoxicity in HeLa cells. Collections of this soft coral from other parts of the world showed different metabolites of this soft coral, e.g., furanocembranoides, which demonstrated antiproliferative activities against the cell lines L-929 and K-562 [8], sesquiterpenes, sterols, and fatty acid derivatives [9,10] all believed to contain medicinal properties. Herein, we report on the isolation and identification of nine compounds from *L. arboreum* collected from Sharm El-Sheikh, Red Sea. The anti-HIV and anti-cancer potential of some of the purified compounds from the soft coral is reported here for the first time.

## 2. Results and Discussion

### 2.1. Bioactivity of Isolated Compounds

When screening several Red sea marine organisms for biological activity, an ethyl acetate fraction of *L. arboreum* demonstrated very strong cytotoxicity in U937 (IC_50_ 6.50 ± 2.3 µg/mL) and moderate cytotoxicity (IC_50_ 28.10 ± 1.2 µg/mL) in HeLa cell lines. The fraction also showed strong HIV-1 PR inhibitory activity (IC_50_ 12 ± 1.3 µg/mL). These results provided justification for further chemical investigation of the lipophilic extract. Activities of the extract were characterized as discussed in Le Roux *et al.*, 2011 [11].

**Figure 1:**
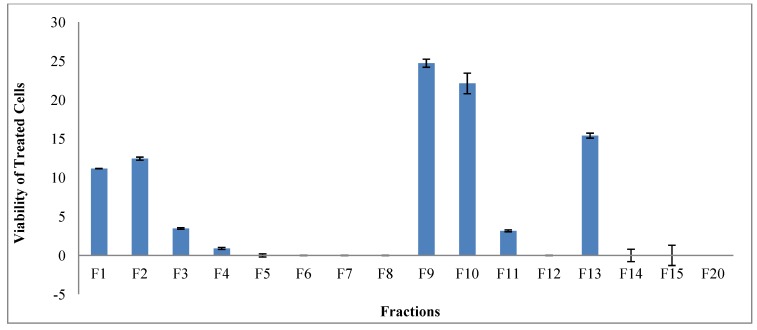
Cytotoxicity of *L. arboreum* main fractions tested in U937 cells at 100 µg/mL.

The chromatographic process yielded 20 major fractions. The cytotoxicity of the extract in U937 cells was used to direct the bioassay-guided fractionation. U937 is one of the most resistant cancer cell lines. Anti-cancer drugs and HIV treatment are related since nucleotide analogs can be used for the treatment of both medical conditions making it possible for the same extracts to contain compounds active against both. Figure 1 shows that, between the twenty fractions, only fractions 5–8, 12, 14, 15, and 20 were 100% cytotoxic in U937 cell line when tested at a concentration of 100 µg/mL. Fractions 16–19 did not show any toxicity in the cells.

**Figure 2:**
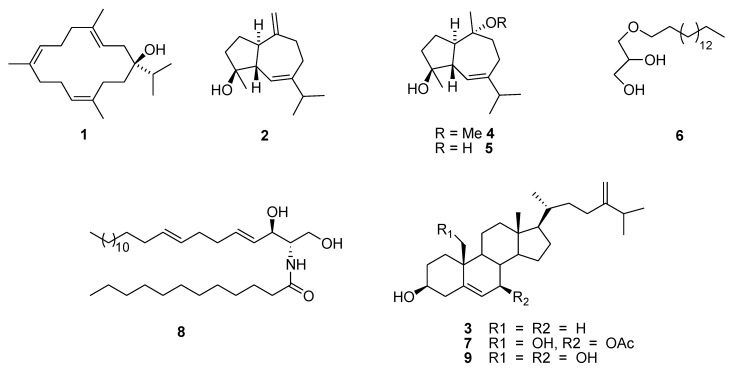
Chemical structure of *L. arboreum* isolated compounds.

Further fractionation and purification of the active fractions resulted in the isolation and identification of nine known compounds (for which the structures are provided in Figure 2) isolated for the first time from this organism sarcophytol M (**1**) [12], alismol (**2**) [13,14], 24-methylcholesta-5,24(28)-diene-3β-ol (**3**) [15], 10-*O*-methyl alismoxide (**4**) [13,16], alismoxide (**5**) [13,14], (*S*)-chimyl alcohol (**6**) [17], 7β-acetoxy-24-methylcholesta-5-24(28)-diene-3,19-diol (**7**) [18], erythro-*N*-dodecanoyl-docosasphinga-(4*E*,8*E*)-dienine (**8**) [19], and 24-methylcholesta-5,24(28)-diene-3β,7β,19-triol (**9**) [20].

Compound **1** known as sarcophytol M (or serratol), was isolated for the first time in a high yield from *Boswellia serrata* [12] and it showed activity against *Trypanosoma brucei* and *Plasmodium falciparum* [16]. Compounds **2**, **4**, and **5** are rare metabolites, identified as active constituents found in extracts from *Alismatis* rhizome [12,14,16]. Compound **2** was found to inhibit the vascular contraction of rabbit thoracic aorta through increasing Ca^2+^ retention [21,22]. It demonstrated antihypertensive potential [23], and showed promising inhibitory effects on INF-γ-induced nitric oxide production in murine macrophage RAW264.7 cells [24]. Compound **3** was previously isolated from the soft coral *Sinularia gibberosa* of Kenting coast, Taiwan but there are no reports on its biological activities. This study is the first report on the isolation of compound **4** and the second report of compounds **2** and **5** from marine resources [8], and it is the first report of the HIV-1 enzyme inhibitory activities of most of these compounds, as well as the cytotoxicity in HeLa and U937 cancer cell lines.

### 2.2. Cytotoxicity

The responses of the isolated compounds were characterized according to P. Prayong *et al.*, (2008) [25], where an IC_50_ value of <5 µg/mL for a pure compound is viewed as strongly cytotoxic. A selectivity index >3 indicates high selectivity [26]. SI values were calculated as follows: IC_50_ of the extract tested in the Vero cell line/IC_50_ of the extract tested in the cancer cell. The inhibitory concentrations and selectivity indices of the isolated nine compounds shown in Table 1 showed that, steroids (7β-acetoxy-24-methylcholesta-5-24(28)-diene-3,19-diol (**7**) and 24-methylcholesta-5,24(28)-diene-3β,7β,19-triol (**9**)) showed the highest cytotoxicity among the isolated compounds. Compound **7** demonstrated strong cytotoxicity and selectivity (IC_50_ 5.3 ± 0.60 µM (4.3 µg/mL), SI 7.2) in HeLa cells and high selectivity with moderate cytotoxicity (IC_50_ 10.6 ± 0.12 µM (7.8 µg/mL), SI 2.9) in U937 cells. Compound **9** showed similar results with strong cytotoxicity (IC_50_ 8 ± 0.5 µM (3.4 µg/mL) but moderate selectivity (SI 1.4)) in HeLa cells and moderate cytotoxicity (IC_50_ 16.4 ± 1.25 µM and moderate selectivity (SI 1.3)) in U937 cells.

**Table 1:** Cytotoxic inhibitory concentrations, selectivity indexes and HIV-1 PR inhibitory concentrations of the nine isolated compounds in different cell lines.

Compound	Cytotoxicity IC_50_ (µM)			SI Values		IC_50_ (µM)
	HeLa	Vero	U937	HeLa	U937	HIV-1 PR
**1**	27.5 ± 0.2	22 ± 0.2	31.7 ± 3.2	0.8	0.75	15.7 ± 0.10
**2**	30 ± 17.2	49	N/T	1.6	N/T	7.2 ± 0.7
**3**	48 ± 8.7	100 ± 1.2	N/T	2.1	N/T	N/T
**4**	38 ± 0.7	49.8 ± 0.5	50 ± 0.23	1.3	1.00	N/T
**5**	>100	>100	>100 ± 0.04	N/A	N/A	N/T
**6**	23.35 ± 5.8	60 ± 1.14	N/T	2.6	N/T	26.6 ± 2.6
**7**	5.3 ± 0.6	31.3 ± 14.03	10.6 ± 0.12	7.2	2.9	4.85 ± 0.18
**8**	38.17 ± 0.7	>100	N/T	2.6	2.95	4.8 ± 0.92
**9**	8 ± 0.5	11.4 ± 0.04	16.4 ± 1.25	1.4	1.3	N/T
Positive control						
Actinomycin D	5.1 ± 0.1	8.8 ± 2.5	1.9 ± 0.865	1.7	4.72	
Acetyl pepstatin						8.5 ± 0.72

N/T: Not tested compounds (inhibitory % < 60% for PR inhibition and <80% cytotoxic).

7β-acetoxy-24-methylcholesta-5-24(28)-diene-3,19-diol (**7**) from the soft coral *Nephthea chabroli* and compound **9** from the soft coral *Litophyton virides* showed sp. comparable cytotoxic activity for tests against different human cancer cell lines; the compounds showed cytotoxicity against human prostate cancer cell line LNCaP with IC_50_ of 15.5 and 4.9 µg/mL [27], A549 cancer cell lines (0.81, 0.69 µg/mL), HT-29 (0.87, 0.72 µg/mL), KB (0.38, 0.58 µg/mL), P-388 (0.42, 0.24 µg/mL) [28] (activities for compound **7** are presented first followed by activity of compound **9**). Activity against *Mycobacterium tuberculosis* was reported for compound **9** [29], as was an immunosuppressive ction [30]. In addition, 24-methylcholesta-5,24(28)-diene-3β,7β,19-triol (**9**) showed preventive action against lipid peroxidation when mixed with different tissue homogenates *in vivo* [31]. The changing of functional groups of compounds **7** and **9**, especially at C-19 in addition to the 5-ene B ring, which is normally observed in an 8β, 9α-half-chair conformations [32], contributed strongly to the final activity and the mode of interaction with the cell membranes. However, there are small differences in the activity of these compounds which stem from the blocking of the hydroxyl group at C-7 (compound **7**) and thus increases the lipophilicity character of the compound. This supports the effect of lipophilicity on the biological activities as it controls the ability of a drug to penetrate various biological membranes, tissues, or barriers and represents a primary factor in controlling the interaction of drugs with biological systems [33].

Moderate cytotoxicity was shown by compound **1** (IC_50_ 27.5 ± 0.2 µM (8.1 µg/mL), SI 0.8). The rest of the compounds showed weak cytotoxicity (>10 µg/mL) compared to Actinomycin D IC_50_s (5.1 ± 0.1 µM for HeLa, 8.8 ± 2.5 µM for Vero, and 1.9 ± 0.87 μM for U937).

### 2.3. Real Time Cell Analysis

**Figure 3:**
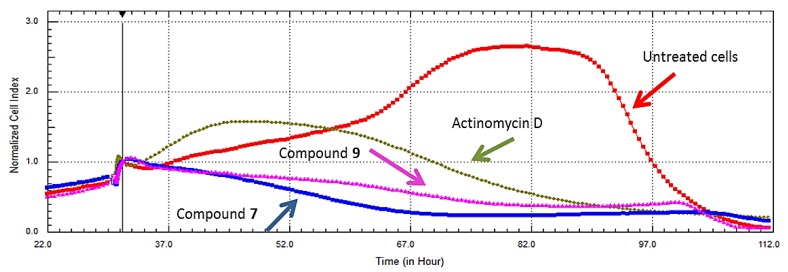
Comparison of RT-CES data of compounds 7β-acetoxy-24-methylcholesta-5-24(28)-diene-3,19-diol (**7**) and 24-methylcholesta-5,24(28)-diene-3β,7β,19-triol (**9**) showing cytotoxicity profiles in HeLa cells. The compounds were more toxic than the positive control, Actinomycin D.

Compound cytotoxicity was investigated further by confirmatory assays in HeLa cells, involving no-label real time cell analysis. Figure 3 shows the real time cell analyses for compounds **7** and **9**, which confirmed their cytotoxic effects when tested at IC_50_s obtained by the end point assay XTT in the same cell line (4.3 µM and 8 µM, respectively).

**Figure 4:**
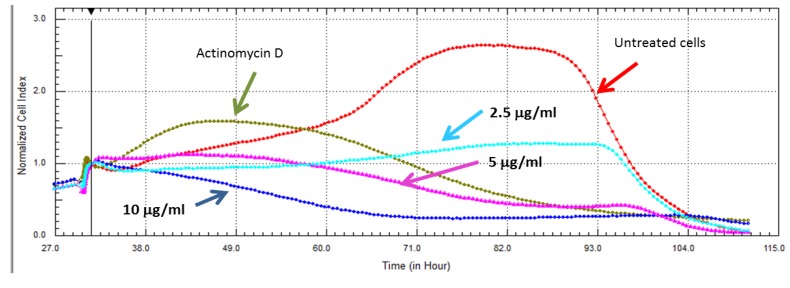
The effect of compound **7** on the proliferation of HeLa cells using RT-CES analysis. The compound displayed a dose dependent cytotoxic tendency at the IC_50_ obtained by *in vitro* viability assay, and twice that amount. The lowest concentration, half of IC_50_ (2.5 µg/mL), displayed cytostatic effects when compared to the untreated cells grown in culture medium only. Actinomycin D (5.6 µM) was used as a positive control for cell death.

Further dose effects of compound **7** in HeLa cells (shown in Figure 4) were evaluated by testing the compound’s cytotoxic effect at 10, 5, and 2.5 µg/mL (13.4, 6.73, 3.36 µM). At 10 and 5 μg/mL, the compound initially underwent an uptake phase and then presented a dose dependent cytotoxic response for the duration of the experiment. While at a lower concentration (2.5 µg), the cells maintained a dose dependent cytostatic response for the duration of the experiment. Cytostatic drugs do not kill cancer cells but instead stop cells from proliferating. This is the first report of the promising selectivity and cytotoxicity of compound **7** for which data was collected using a viability dye and confirmed with RT-CES; this holds a great promise for use of this compound in chemo-preventive and chemotherapeutic strategies.

Alismol (**2**) exhibited moderate cytotoxicity while Alismoxide (**5**) showed no toxicity *in vitro*, but data collected with xCELLigence showed the cytostatic effect of the compounds when tested at 30 µg/mL and 100 µg/mL.

**Figure 5:**
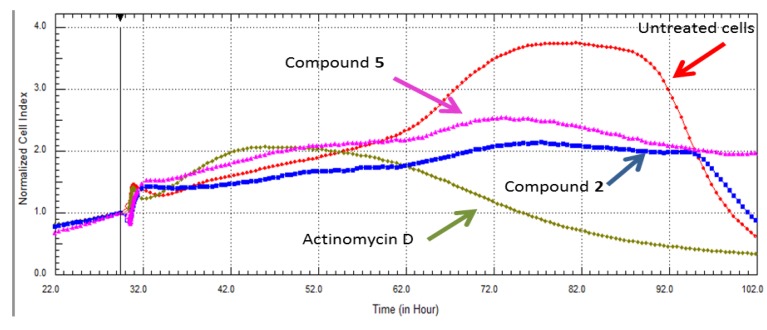
The effect of compound **2** at 16 μg/mL and compound **5** at 60 μg/mL in HeLa cells were compared to the untreated cells (culture media only) and the positive control Actinomycin D. The compounds demonstrated cytostatic behavior (in the presence of these compounds, cells did not proliferate, nor did they die).

As shown in Figure 5, it is clear that the compounds inhibited 50% of the cell proliferation compared to the untreated cells. The RT-CES diagram shows uptake of the compounds by the cells during the first h of incubation, later untreated cells proliferate (higher cell indices—CI) while cells incubated with compounds **2** and **5** do not show an increased CI, which can be interpreted as these compounds being cytostatic (anti-proliferative). This result supports the fact that RT-CES data is more reliable as it is not subjected to the shortcomings of the end point assays, such as sensitivity to environmental conditions or dependence on the cells’ metabolism of formazan [34].

### 2.4. Inhibitory Activities of HIV-1 Enzymes

**Figure 6:**
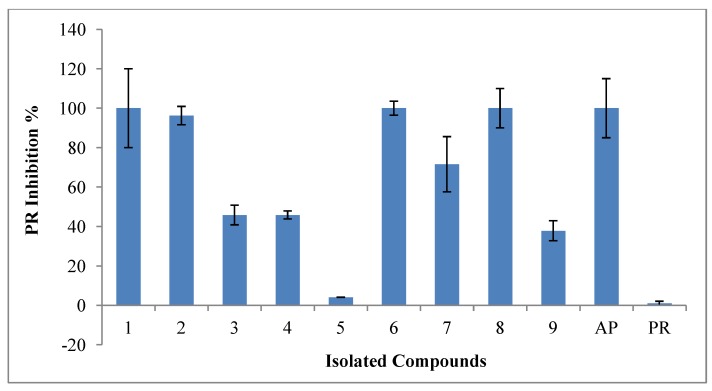
Protease inhibition of *L. arboreum* isolated compounds at 100 µg/mL. Fractions are indicated by number while the known inhibitor used as positive control, acetyl pepstatin is abbreviated AP.

The isolated compounds were screened for their inhibitory activity against HIV-1 RT and HIV-1 PR enzymes at 100 µg/mL. None of the compounds inhibited the HIV-1 RT enzyme, while, as shown in Figure 6 for HIV-1 PR, compounds Sarcophytol M (**1**), Chimyl alcohol (**6**) and and Erythro-*N*-dodecanoyl-docosasphinga-(4*E*,8*E*)-dienine (**8**) showed 100% inhibition. Compound **2** showed inhibition of 96.2% while 7 demonstrated moderate inhibition of 71.5%.

The fifty percent inhibitory concentrations (IC_50_s) were determined only for the compounds that inhibited more than 50% of the enzyme activity. The efficacy of the compounds was defined as potent for compounds with IC_50_ < 5.6 µM as described by Cheenpracha *et al.*, 2006 [35], weak compounds with IC_50_s > 10 µM as described in Marastoni *et al.*, 1997 [36].

The detailed IC_50_ results expressed in Table 1, showed potent inhibitory activity of HIV-1 PR by compounds **7** (4.85 ± 0.18 µM), and 8 (IC_50_ 4.80 ± 0.92 µM) compared to the positive control, which had IC_50_ of 8.5 ± 0.72 µM. Compound **2** showed moderate inhibitory activities with IC_50_ (7.2 ± 0.70 µM).

Sarcophytol M (**1**) and the Chimyl alcohol (**6**) showed weak inhibitory activities (15.7 ± 0.103 µM and 26.6 ± 2.6 μM).

**Figure 7:**
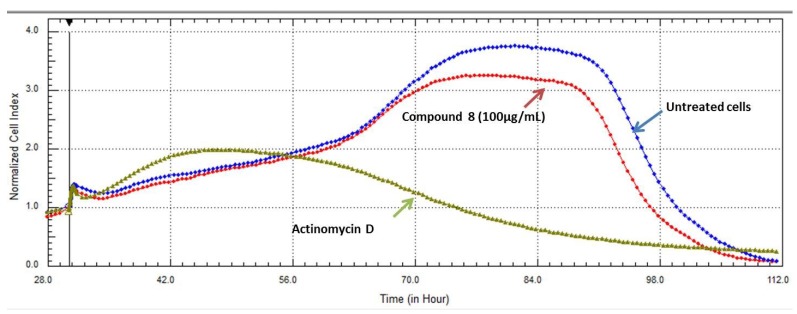
The effect of the active compound **8** on the proliferation of HeLa cells using RT-CES analysis. Cells treated with a high concentration (100 µg/mL) of compound **8** showed low level cytotoxicity compared with the untreated cells grown in culture medium only and the positive control Actinomycin D.

The cleavage of host cell proteins by HIV-1 PR results in cell death via several necrotic and apoptotic pathways, possibly leading to depletion of CD4 + T-cells. Thus, protease inhibitors are particularly useful, not only because they inhibit viral replication but also because they rescue host immune cells [37]. Potent PR inhibition were found in our bioassay for some of the tested compounds, compound **7** demonstrated activity at (IC_50_ 4.85 µM). At this concentration, the compound showed 50% cell death in HeLa and 20% cell death in U937 cells while it had no effect in Vero (normal cells) as the IC_50_ in these cells was 31.3 µM. On the other hand it was very interesting to find that, compound **8** (erythro-*N*-dodecanoyl-docosasphinga-(4*E*,8*E*)-dienine) was not toxic in any of the tested cell lines with IC_50_s 71 µM in HeLa, >100 µM in Vero cell lines and >100 µM in U937 and its effects in HeLa cells was confirmed with real time cell analysis as shown in Figure 7. Cells treated with a very high concentration (100 µg/mL) of compound **8** were only affected at long incubation times compared to the untreated cells. The mechanism of action of compound **8** is not known yet. The compound could be a very promising candidate for designing new lead compounds targeting HIV-1 infection, as the lipid rafts of the host cells primarily consist of sphingolipids and cholesterol and these have been implicated in the infectious route of HIV-1 entry. As lipid rafts are recognized sites for budding and entry of HIV-1, modulating the composition/structure of lipid rafts can influence the life cycle of HIV-1 inhibiting its replication [38].

### 2.5. Molecular Docking

**Figure 8:**
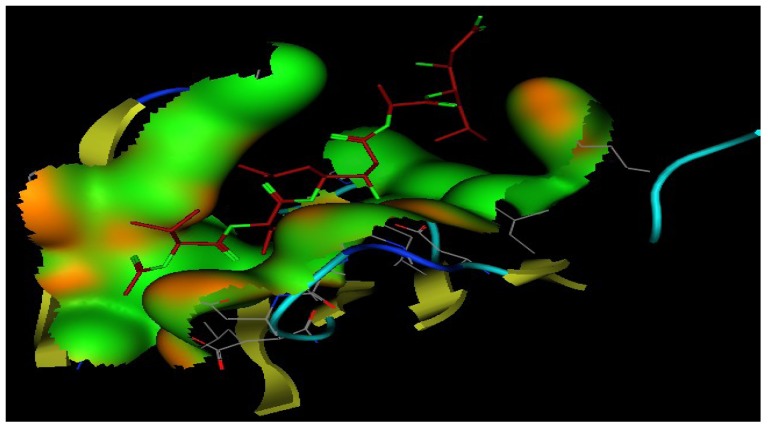
3D Stereo-diagram of the ligand “acetyl pepstatin” combined with the HIV-1 protease receptor (5HVP) active site. The green color shows the lipophilic nature of the pocket.

In the 3D stereo-diagram of acetyl pepstatin bound to the HIV-1 protease active site shown in Figure 8, the X-ray structure clearly reveals that most of the amino acid moieties surrounding the inhibitor are hydrophobic. The figure was based on the X-ray crystal structure 5HVP available from the Protein Data Bank. The moieties that make up these pockets (among others) are Asp 25, Gly 27, Asp 29, Gly 48, Ile 50, and Thr 80, in each monomer, and as seen from the green color in the figure, which indicate the hydrophobic nature of the receptor pocket.

**Figure 9:**
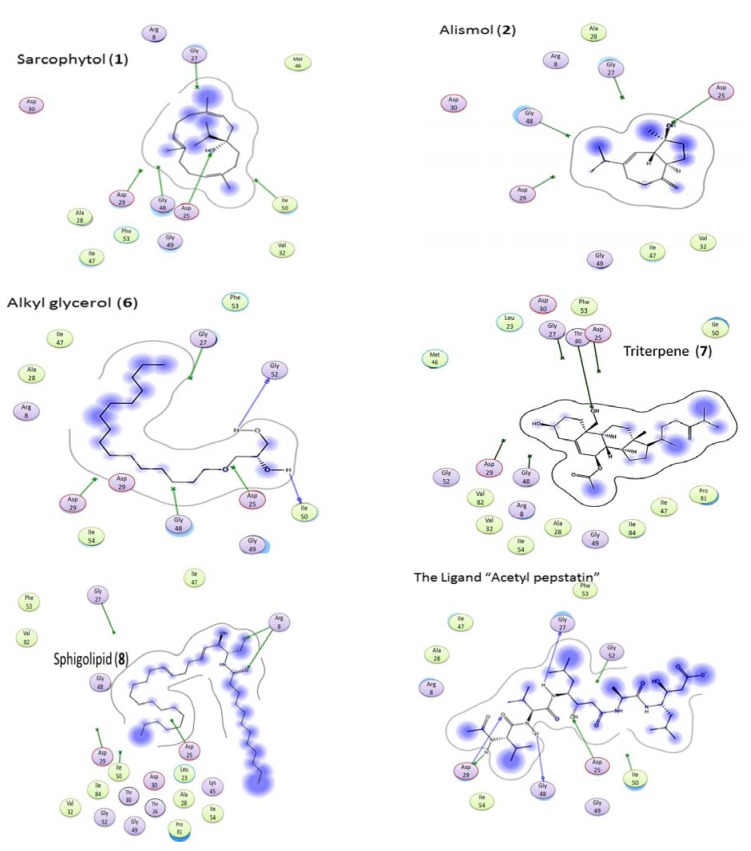
Interaction of bioactive compounds with different amino acid moieties in HIV-1 protease receptor (5HVP) active site.

Figure 9 shows the interaction of the ligand and the bioactive compounds (**1**, **2**, **6**, **7**, and **8**). The ligand (acetyl pepstatin) interact through different hydrogen bonds with different amino acid moieties as shown in Table 2 and Figure 9 and scoring −24.79. In addition, Table 2 also shows the interaction mode of bioactive compounds in the active site with different amino acid moieties with the corresponding measured bond distances. Interestingly, the compound containing an alkyl and an alkenyl chain gave a higher score, e.g., compound **6** (with long hydrocarbon chain) scored −17.16 followed by compound **8** (containing two fatty chains) scored −15.92 and compound **1** (with a lipophilic backbone containing only a hydroxyl group) scored −14.44. In addition, this trend is very clear from the higher activity of compound **7** when compared to compound **9**, which had only an extra acetyl group and a more lipophilic character. The data above indicated the importance of the hydrophobic part of different ligands when interacting in the binding pocket, and confirms the contribution of the hydrophobicity of inhibitors on HIV protease. Surprisingly, most of the nucleoside drugs in use are hydrophilic. Results presented here support the existing treatment strategies of using a combination of (hydrophilic and hydrophobic) drugs in the treatment of HIV.

**Table 2:** Hydrogen bond types and scores of bioactive compounds interacting with HIV-1 protease receptor (5HVP) active site.

Ligand/Compound	Residue	Type	Distance	Score
Ligand	ASP 25	H-don	1.99	−24.79
	ASP 25	H-don	2.72	
	GLY 27	H-don	2.16	
	ASP 29	H-don	1.95	
	GLY 48	H-don	1.94	
	ASP 25	H-acc	1.99	
	ASP 25	H-acc	2.72	
	ASP 29	H-acc	2.81	
**1**	ASP 25	H-don	2.45	−14.44
	ASP 25	H-acc	2.45	
**2**	ASP 25	H-don	2.37	−11.14
	ASP 25	H-acc	2.37	
**6**	ILE 50	H-don	1.50	−17.16
	GLY 52	H-don	1.65	
**7**	THR 80	H-don	2.75	−18.36
	THR 80	H-acc	2.75	
**8**	ARG 8	H-Acc	2.61	−15.92
	ARG 8	H-acc	3.01	

## 3. Experimental Section

### 3.1. General Experimental Procedures

The NMR spectra were recorded on Bruker spectrometer at 400 MHz for 1H and 100 MHz for 13C, and/or Varian “Mercury” 200 MHz, for ^1^H and 50 MHz for ^13^C in CDCl3 with TMS as internal standard. Chemical shifts are given in δ (ppm) and coupling constants in Hertz. EIMS were recorded by a Shimadzu Qp-2010 plus. Cytotoxicity plates red by BIOTEK Power-Wave XS multiwell plate reader was used for the cytotoxicity assays. Pre-coated Si gel plates (Merck, Johannesburg, South Africa, Kieselgel 60 F254, 0.25 mm) were used for analytical TLC analyses. Silica gel 60 (Merck, Johannesburg, South Africa, 230–400 mesh) and Sephadex LH-20 from Pharmacia Fine Chemicals AB (Uppsala, Sweden) were used for column chromatography. Methanol, Dichloromethane, Ethyl acetate and DMSO, were obtained from Merck (Johannesburg, South Africa). All cell lines, media, trypsin-EDTA, fetal bovine serum (FBS), and antibiotics (penicillin, streptomycin, and fungizone) were supplied by Highveld Biological (Pty) Ltd. (Modderfontein, Johannesburg, South Africa). A puriﬁed recombinant HIV1-RT (Merck, Darmstadt, Germany) was used with the cell free reverse transcriptase colorimetric kit (Roche Diagnostics, Mannheim, Germany). The HIV-1 protease enzyme (Bachem Bioscience Inc., King of Prussia, PA, USA) and the substrate (a synthetic peptide that contains a cleavage site Tyr-Pro for HIV protease, as well as two covalently modiﬁed amino acids for the detection of cleavage) were used.

### 3.2. Animal Material

The soft coral *L. arboreum* was collected at Sharm El-Sheikh (Red Sea, Egypt), in May 2010, at a depth of 2–3 m, the tissue was kept in MeOH and extracted *in situ*. The material was collected and identified by Mona Ellithey, co-author of this article.

### 3.3. Purification of the Active Constituents

The sliced bodies of *Litophyton arboreum* (2 kg, fresh material) were blended with MeOH, after filtration the residue was washed twice with fresh MeOH. The total extracts were concentrated under a vacuum, and partitioned between EtOAc and H_2_O. The solvent-free EtOAc fraction (25 g) was subjected to column chromatography on silica gel and eluted with mixtures of increasing polarity from hexane and EtOAc (0%–100%) to yield 43 fractions, which were pooled together according to the TLC profile to twenty main fractions.

The twenty fractions (1–20) were tested for their cytotoxic activity on U937 cell line at 100 µg/mL. The main fractions 5–8, 11, 12, 14, 15, and 20 showed potent cytotoxicity (Figure 1) and were subjected to chromatographic purification. Fraction 5 was chromatographed (on silica gel using hexane/EtOAc (98:2)) producing compound **1** (60 mg). Fraction 6 under the same conditions yielded pure **2** (750 mg). Fraction 7 was chromatographed (on silica gel using hexane/EtOAc (95:5)) producing compound **3** (40.2 mg). Fractions 8 was chromatographed [on silica gel] and produced transparent oily pure compound **4** (650 mg). Fraction 11 on standing at room temperature for three weeks produced crystals, which were washed with a mixture of hexane and EtOAc and produced pure compound **5** (1.2 g). Fraction 12 was chromatographed (on silica gel using gradient mixture of hexane/EtOAc (80:20 to 70:30)) producing compound **6** (200 mg). Fraction 14 was chromatographed (on silica gel using mixture of hexane/EtOAc (75:25)) producing compounds **7** (50 mg) and **8** (75 mg). Fraction 20 was chromatographed (on silica gel using hexane/EtOAc (70:30)) producing compound **9** (20 mg).

Sarcophytol M (**1**): Oil, 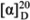 +34 (*c*.0.76 CHCl_3_,); ^1^H NMR, (200 MHz, CDCl_3_) δ_H_ 5.25 (1H, t, *J* = 6.8 Hz, H-3); 4.91 (1H, t, *J* = 7.8 Hz, H-11); 4.89 (1H, t, *J* = 6.8 Hz, H-7); 1.61 (3H, s, Me-20); 1.59 (3H, m, H-19), 1.56 (3H, s, Me-18); 0.96, 0.94 (3H each, d, *J* = 6.8; Me-16, 17). ^13^C-NMR (50 MHz, CDCl_3_) δ_C_ 136.7 (C-4), 135.5 (C-12), 133.3 (C-8), 125.9 (CH, C-7), 123.2 (CH, C-11), 120.9 (CH, C-3), 76.9 (C-1), 39.9 (CH_2_, C-9), 39.5 (CH_2,_ C-5), 35.0 (CH_2_, C-14), 34.8 (CH_2_, C-2), 34.6 (CH, C-15), 33.5 (CH_2_, C-13), 24.8 (CH_2_, C-6), 23.7 (CH_2_, C-10), 16.9 (CH_3_, C-17), 16.6 (CH_3_, C-16), 16.2 (CH_3_, C-20), 15.4 (CH_3_, C-19), 15.2 (CH_3_, C-18) [12].

Alismol (**2**): Oil; 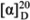 +4.9 (*c*.0.2 CHCl_3_,); ^1^H NMR (200 MHz, CDCl_3_): δ_H_ 5.56 (1H, s, H-6), 4.69,4.64 (1H each, s, H2-14); 1.17 (3H, s, Me-15), 0.93, 0.92 (3H each, d, *J* = 6.6 Hz, Me-13, 14); ^13^C NMR (50 MHz, CDCl_3_): δ_C_ 153.9 (C-10), 149.3 (C-7), 121.4 (CH, C-6), 106.4(CH_2_, C-15), 80.4 (C-4), 54.6 (CH, C-1), 47.0 (CH, C-5), 40.0 (CH_2_, C-3), 37.3 (CH, C-11), 36.9 (CH_2_, C-9), 29.8 (CH_2_, C-8), 24.6 (CH_2_, C-2), 23.8 (CH_3_, C-14), 21.3 (CH_3_, C-13), 21.1(CH_3_, C-12) [13,14].

24-Methyl-cholesta-5,24(28)-diene-3β-ol (**3**): 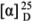 +7.2° (*c* 0.3 CHCl_3_); ^1^H NMR (400 MHz, CDCl_3_) δ_H_ 0.65 (3H, br s, H-18), 0.92 (3H, d, *J*= 6.18, H-21), 0.97 (3H, br s, H-19), 1.00 (3H, d, *J* = Hz, H-27), 0.98, 1.00 (3H, d, J= Hz, H-26), 3.51 (1H, br s, H-3), 4.63 (1H, s, H-28a), 4.69 (1H, s, H-28b), 5.32 (1H, s, H-6). ^13^C NMR (100 MHz, CDCl_3_) data: δ_C_ 157.3 (C-24), 141.2 (C-5), 122.1 (CH, C-6), 106.4 (CH_2_, C-28), 72.1 (CH, C-3), 57.2 (CH, C-14), 56.4 (CH, C-17), 50.5 (CH, C-9), 42.6 (C-13), 42.8 (CH_2_, C-4), 40.1 (CH_2_, C-12), 37.7 (CH_2_, C-1), 36.2 (C-10), 35.7 (CH, C-20), 35.1 (CH_2_, C-23), 34.2 (CH, C-25), 32.3 (CH, C-8), 32.0 (CH_2_, C-7), 32.0 (CH_2_, C-2), 31.4 (CH_2_, C-22), 28.6 (CH_2_, C-16), 24.7 (CH_2_, C-15), 22.4 (CH_3_, C-27), 22.3 (CH_3_, C-26), 21.5 (CH_2_, C-11), 19.8 (CH_3_, C-19), 19.1 (CH_3_, C-21), 12.3 (CH_3_, C-18) [15].

10-*O*-Methyl alismoxide (**4**): Oil, 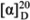 +5.9° (*c* 0.3 CHCl_3_). ^1^H NMR (200 MHz, CDCl_3_) δ_H_ 5.41 (1H, br s, H-6), 3.12 (OMe); 1.13, 1.14 (3H each, s, Me-14, 15); 0.93, 0.90 (3H each, d, *J* = 6.6 Hz, Me-12, 13); ^13^C NMR (50 MHz, CDCl_3_): δ_C_ 149.5(C-7), 121.2 (CH, C-6), 80.1 (C-4), 79.2(C-10), 50.0 (CH, C-5), 48.6 (OCH_3_), 47.8 (CH, C-1), 40.4 (CH_2_, C-3), 37.1(CH, C-11), 35.4(CH_2_, C-9), 24.5(CH_2_, C-8), 22.3(CH_3_, C-14), 21.6 (CH_2_, C-2), 21.5 (CH_3_, C-13), 21.2 (CH_3_, C-12), 17.9 (CH_3_, C-15) [13,16].

Alismoxide (**5**): Colorless crystals, mp 138–141 °C; 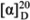 +9.3 (*c* 0.9 CHCl_3_,) ^1^H NMR (400 MHz, CDCl_3_) δ_H_ 5.44 (1H, br d, *J* = 3.0 Hz, H-6), 0.98, 1.0 (3H each, d, *J* = 6.9 Hz, H-12, 13), 1.25, 128 (3H each, s, H-14,15);^13^C NMR (100 MHz, CDCl_3_): δ_C_ 149.4(C-7), 121.3 (CH, C-6), 80.0 (C-4), 75.2 (C-10), 50.5 (CH, C-1), 50.1 (CH, C-5), 42.5 (CH_2_, C-9), 40.3 (CH_2_, C-3), 37.2 (CH, C-11), 25.0 (CH_2_, C-8), 22.4 (CH_3_, C-14), 21.4 (CH_2_, C-2), 21.3 (CH_3_, C-15), 21.2 (CH_3_, C-13), 21.1 (CH_3_, C-12).

Chimyl alcohol (**6**): 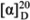 +7.2° (*c* 0.3 CHCl_3_); ^1^H NMR (CDCl_3_, 200 MHz) δ_H_, 3.83 (1 H, m, H-2), 3.72, 3.64 (2H, m, H-1), 3.45 (4H, m, H-1′, 3), 1.52 (2H, m, 2′-H2), 1.26 (26H, br s, CH_2_), 0.84 (3H, t, *J* = 6.6 Hz, CH_3_); ^13^C NMR (50 MHz, CDCl_3_): δ_C_ 72.4 (CH_2_-1′), 71.8 (CH_2_-1), 70.5 (CH-2), 64.2 (CH_2_-3), 31.9 (CH_2_-13′), 29.7-29.3 (CH_2_′s), 26.0 (CH_2_-3′), 22.7 (CH_2_-14′), 14.1 (CH_3_). HRESIMS: 317.3045 [M + H]^+^ corresponding to C_19_H_41_O_3_, calculated for 317.3057 [13,14].

7β-Acetoxy-24-methyl-cholesta-5,24(28)-diene-3β,19-diol (**7**): Amorphous powder, 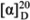 +6.7° (*c* 0.5 CHCl_3_); ^1^H NMR (400 MHz, CDCl_3_): δ_H_ 5.56 (1H, t, *J* = 2 Hz, H-6), 4.95 (1H, dt, *J* = 2.2, 2.2, 8.5 Hz, H-7α), 4.70 (1H, t, *J* = 1.3 Hz, H_A_-28), 4.64 (1H, dd, *J* = 1.3 and 1.7 Hz, H_B_-28), 3.87 (1H, d, *J* = 11.5 Hz, H_A_-19), 3.64 (1H, d, *J* = 11.5 Hz, H_B_-19), 3.59 (1H, tt, *J* = 11.2 4.7 Hz, H-3α), 2.38 (1H, ddd, *J* = 13.5, 4.7, 2.4 Hz, H-4α), 2.01 (3H, s, OAc), 1.01 (3H, d, *J* = 6.8 Hz, Me-26), 1.00 (3H, d, *J* = 6.8 Hz, Me-27), 0.93 (3H, d, *J* = 6.8 Hz, Me-21), 0.73 (3H, s, Me-18). ^13^C NMR (100 MHz, CDCl_3_): δ_C_ 171.4 (Me*C*OO-), 156.7 (C-24), 140.1 (C-5), 126.6 (CH, C-6), 106.0 (CH_2_, C-28), 75.2 (CH, C-7), 70.8 (CH, C-3), 62.8 (CH_2_, C-19), 56.5 (CH, C-14), 55.2 (CH, C-17), 48.5 (CH, C-9), 43.1 (C-13), 41.6 (CH_2_, C-4), 41.4 (qC, C-10), 39.7 (CH_2_, C-12), 37.8 (CH, C-8), 35.6 (CH, C-20), 34.6 (CH_2_, C-22), 33.7 (CH, C-25), 33.1 (CH_2_, C-1), 31.7 (CH_2_, C-2), 30.9 (CH_2_, C-23), 28.3 (CH_2_, C-16), 24.9 (CH_2_, C-15), 22.0 (CH_3_, C-26), 21.8 (CH_3_, C-27), 21.7 (CH_3_, *C*H_3_COO), 21.6 (CH_2_, C-11), 18.7 (CH_3_, C-21), 12.1 (CH_3_, C-18) [18].

Erythro-*N*-dodecanoyl-docosasphinga-(4*E*,8*E*)-dienine (**8**): ^1^H NMR (400 MHz, CDCl_3_): δ_H_ 6.25 (1H, d, *J*_NH,2_ = 7.6 Hz; H-NH), 5.78 (1H, dtd, *J* = 15.4, 6.4, 1.2 Hz; H-5), 5.54 (1H, ddt, *J* = 15.4, 6.3, 1.3 Hz; H-4), 5.42 (1H, dt, *J* = 15.3, 6.3 Hz; H-9), 5.36 (1H, dt, *J* = 15.3, 5.9 Hz; H-8), 4.32 (1H, br, apparently triplet, *J* = 4.9, 6.3, 1.2 Hz; H-3 (please note that the *J* values were measured in the other coupled protons)), 3.95 (1H, dd, *J* = 11.0, 3.2 Hz; H_A_-1), 3.90 (1H, dddd, *J* = 3.2, 3.2, 4.9 Hz, *J*_2,NH_ = 7.6 Hz; H-2), 3.70 (1H, dd *J* = 11.0, 3.2 Hz; H_B_-1), 2.23 (2H, t, *J* = 7.4 Hz; 2H-2′), 2.12 (2H, m; 2H-6), 2.08 (2H, m; 2H-7), 1.96 (2H, q, *J* = 6.4, 6.4 Hz; 2H-10), 1.64 (4H, m; 2H-3′ and 2H-of other position), 1.25 (-(CH_2_)_n_-), 0.88 (6H, t, *J*_vic_ = 7.0 Hz; Me-22 and Me-16′); ^13^C NMR (100 MHz, CDCl_3_): δ_C_ 173.9 (C-1′), 133.5 (CH, C-5), 131.6 (CH, C-9), 129.2 (CH, C-4), 129.0 (CH, C-8), 74.7 (CH, C-3), 62.5 (CH_2_, C-1), 54.4 (CH, C-2), 36.8 (CH_2_, C-2′), 32.6 (CH_2_, C-10), 32.3–31.9 (all CH_2_), 29.7–29.1 (all CH_2_), 25.8 (2CH_2_, C-3′ and other CH_2_), 22.7 (2CH_2_, C-21 and C-15′), 14.1 (2CH_3_, C-22 and C-16′). HRESIMS: found 536.5040 [M + H]^+^, calculated for C_34_H_66_NO_3_ 536.50458 [19].

24-Methyl-cholesta-5,24(28)-diene-3β,7β,19-triol (**9**): Amorphous powder, 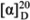 +5.2° (*c* 0.2 MeOH). ^1^H-NMR [200 MHz, CDCl3/CD3OD (1:3)] δ_H_ 5.44 (1H, s, H-6), 4.67, 4.61 (1H each, br s, H2-28) 3.54, 3.80 (1H each, d, *J* = 11.8 Hz, H-19), 3.60 (1H, br s, H-7), 3.42 (1H, m, H-3), 0.94 (6H, d, *J* = 7.0 Hz, Me-26, 27), 0.92 (3H, d, *J*= 6.6, Me-21), 0.72 (3H, br s, Me-18),. ^13^C-NMR [50 MHz, CDCl3/CD3OD (1:3)] δ_C_ 157.7 (C-24), 140.0 (C-5), 131.0 (CH, C-6), 106.9 (CH_2_, C-28), 73.3 (CH, C-7), 72.0 (CH, C-3), 63.4 (CH_2_, C-19), 58.6 (C-14), 56.8 (CH, C-17), 49.8 (CH, C-9), 44.3 (C-10), 42.7 (CH_2_, C-4), 42.4 (CH, C-8), 42.4 (C-13), 41.5 (CH_2_, C-12), 37.0 (CH, C-20), 36.0 (CH_2_, C-22), 34.9 (CH, C-25), 34.0 (CH_2_, C-1), 32.6 (CH, C-2), 32.1 (CH_2_, C-23), 29.7 (CH_2_, C-16) 27.2 (CH_2_, C-15), 22.9 (CH_2_, C-11), 22.5 (CH_3_, C-26), 22.3 (CH_3_, C-27), 19.3 (CH_3_, C-21), 12.6 (CH_3_, C-18) [20].

### 3.4. HIV-1 Direct Enzyme Assays

Reverse transcriptase (RT) inhibitory activity of the crude extracts against a puriﬁed recombinant HIV1-RT (Merck, Darmstadt, Germany) was determined by using the Roche Diagnostics (Mannheim, Germany) colorimetric kit. The assay was performed as previously described [34].

HIV-1 protease enzyme was purchased from Bachem Bioscience Inc. King of Prussia, PA, UK and the substrate (a synthetic peptide that contains a cleavage site Tyr-Pro for HIV protease as well as two covalently modiﬁed amino acids for the detection of cleavage). The assay was performed according to procedures by Lam *et al.*, 2000 [39], in black 96 well assay plates obtained from Corning Incorporated, (Corning, New York, NY, USA). The fluorescence intensity was measured at an excitation wavelength of 355 nm and an emission wavelength of 460 nm using a synergy microplate spectrofluorometer (BioTek, Analytical & Diagnostic products, South Africa). Acetyl pepstatin (AP) was used as a positive control for HIV-1 PR inhibition. DMSO was included in the enzyme control (1% DMSO). Enzyme remains active after this addition as observed from the fluorescent values obtained. The percentage inhibition was calculated based on the formula: 100 − [(Test reagent RFU − background RFU)/(untreated control RFU − blank) × 100] where RFU = relative fluorescence units.

All the isolated compounds were screened at 100 µg/mL for inhibition of HIV-1 enzymes. IC_50_ values of the most active extracts were calculated and compared to known HIV-1 PR and HIV-1 RT inhibitors as well as the untreated enzymes activities. All the experiments were repeated three times in order to ensure the precision and accuracy of the data.

### 3.5. Cytotoxicity

All cell lines, media, trypsin-EDTA, fetal bovine serum (FBS), phosphate buffer saline (PBS) and antibiotics were supplied by Highveld Biological (Pty) Ltd. (Modderfontein, Johannesburg, South Africa).

HeLa, U937, and Vero cell lines were maintained in culture flasks containing Eagle’s Minimum Essential Medium supplemented with 10% heat-inactivated FBS and 1% antibiotics (100 U/mL penicillin, 100 μg/mL streptomycin, and 250 μg/mL fungizone). The cells were grown at 37 °C in a humidified incubator set at 5% CO_2_. Cells were subcultured after they formed a monolayer on the flask. The cells were detached by treating them with trypsin-EDTA (0.25% trypsin containing 0.01% EDTA) for 10 minutes and then by adding complete medium to inhibit the reaction. Cytotoxicity was measured by the XTT method using the Cell Proliferation Kit II. The method described by Zheng *et al.* [40] was used to perform the assay. Cells were seeded (100 μL) in a 96-well microtiter plate (concentration 1 × 10^5^ cells/mL). The plate was then incubated for 24 h at 37 °C and 5% CO_2_ to allow the cells to attach to the bottom of the wells. The compounds were each prepared to a stock solution of 20 mg/mL and added to the microtiter plate. Serial dilutions were made to range from a concentration of 100 μg/mL–0.7 μg/mL for each compound. The microtiter plate was incubated for a further 72 h. The control wells included vehicle-treated cells exposed to 2% DMSO and the positive control Actinomycin D, with concentrations ranging between 0.5 μg/mL and 0.002 μg/mL. After the 72 h incubation period, the XTT reagent (50 μL) was added to a final concentration of 0.3 mg/mL and the plate was then further incubated for another 2 h. After the incubation the absorbance of the color complex was read at 490 nm with a reference wavelength set at 690 nm. Mean IC_50_ is the concentration of compound, which reduces cell growth by 50% under the experimental conditions and is the average of at least three independent reproducible statistically significant measurements. The IC_50_ values were reported at ±95% confidence intervals (±95% CI). The results were analyzed using the GraphPad Prism 4 program.

### 3.6. Real-Time Cell Electronic Sensing (RT-CES) xCELLigence

The xCELLigence system was used according to the instructions of the supplier (Roche Diagnostics, Mannheim, Germany and ACEA Biosciences, San Diego, CA, USA). HeLa cell line, media, trypsin-EDTA, fetal bovine serum (FBS), phosphate buffer saline (PBS), and antibiotics were supplied by Highveld Biological (Pty) Ltd. (Modderfontein, Johannesburg, South Africa).

The xCELLigence system was used according to the instructions of the supplier (Roche Diagnostics, Mannheim, Germany and ACEA Biosciences, San Diego, CA, USA). [41].

The electronic impedance of sensor electrodes that connect the E plate to the instrument is measured to allow monitoring and detection of physiological changes of the cells on the electrodes. The impedance measured between electrodes in an individual well depends on electrode geometry, ion concentration in the well and whether or not cells are attached to the electrodes. In the absence of cells, electrode impedance is mainly determined by the ion environment, both at the electrode/solution interface, and in the bulk solution. In the presence of cells, cells attached to the electrode sensor surfaces will act as insulators and thereby alter the local ion environment at the electrode/solution interface, leading to an increase in impedance [41]. Thus, the more cells that are growing on the electrodes, the larger the value of electrode impedance. This assay has been described for the measurement of cytotoxicity [42,43] and can be used to determine other cellular parameters such as cell proliferation, cytotoxicity start time, cell recovery, and cell response patterns [44] in real time.

HeLa cells were grown and expanded in tissue-culture flasks. After reaching ~75% confluence, the HeLa were washed with PBS, and afterwards detached from the flasks by a brief treatment with trypsin. Subsequently, 50 µL of cell culture media at room temperature was added into each well of E-plate. After this the plate was connected to the system and checked in the cell culture incubator for proper electrical-contacts and the background impedance was measured over 24 h. Meanwhile, the cells were re-suspended in cell culture medium and adjusted to 2 × 10^5^, 1 × 10^5^, 5 × 10^4^, and 2.5 × 10^4^ cells/mL. One-hundred microliters of each cell suspension was added to the 50 µL medium containing wells on the E-plate, in order to determine the optimum cell concentration. After 30 min incubation at room temperature, E-plates were placed into the cell culture incubator. Finally, adhesion, growth, and proliferation of the cells was monitored every 30 min for a period of up to 18 h via the incorporated sensor electrode arrays of the E-Plate. The electrical impedance was measured by the RTCA-integrated software of the xCELLigence system as a dimensionless parameter termed Cell index (CI). After determining the optimal concentration for HeLa cells proliferation, cells at the optimal concentration were seeded in 100 µL in each well of the E-plate, the proliferation, attachment, and spreading of the cells was monitored every 30 min by the xCELLigence system. Approximately 30 h after seeding, when the cell index was 1.5 (in the log growth phase), cells were then treated with the compounds at the IC_50_ obtained by XTT. The positive control Actinomycin D, with concentrations ranging between 0.5 μg/mL and 0.002 μg/mL, was also tested and just cells growing in the culture medium were included as (untreated cells) or negative control. All experiments were run for 72 h after adding the compounds. All calculations were obtained using the RTCA-integrated software of the xCELLigence system.

### 3.7. Molecular Docking

For the purpose of lead optimization and to find out the interaction between the compound and the HIV-1 protease receptor, molecular modeling calculations and local docking were done using MOE. This was done to evaluate the binding free energies of this inhibitor into the target HIV-1 protease receptor, and to find out interactions between ligand and receptor, also, to compare affinities of the isolated bioactive compounds to the target HIV-1 protease receptor. For the docking calculations, the protein structure (PDB code: 5HVP) [45] was first separated from the inhibitor molecule and refined using molecular minimization with added hydrogen.

Docking calculations were carried out using standard default variables for the MOE program. The binding affinity was evaluated by the binding free energies (S-score, kcal/mol), hydrogen bonds, and RMSD values. The compounds were docked into the same groove of the binding site of the native co-crystallize ligand. The Dock scoring was performed with the London dG scoring function and has been enhanced using two different refinement methods, which were capable of sampling conformational changes in the backbone structure. We allowed rotatable bonds; the best 10 poses were retained and analyzed for the binding poses best score. Energy minimization was done through Force-field MMFF94x Optimization using gradient of 0.0001 for determining low energy conformations with the most favorable (lowest energy) geometry.

## 4. Conclusions

In this study, we report for the first time, the isolation, identification and biological evaluation of metabolites from *L. arboreum* collected from Sharm El sheikh (Red Sea). *L. arboreum* extract collected from the southern part of the Red Sea showed more promising cytotoxic metabolites than that of the same organism collected from the South China Sea [10]. A possible reason for this could be spatial and temporal variations in secondary metabolites from organisms from different geographical regions. Potencies of the metabolites produced by an organism may also differ based on the stress experienced by the organism in a particular environment [46]. This report is also the first to provide data on *L. arboreum* extract and the purified compounds demonstrating anti HIV-1 activities where some of the isolated metabolites exhibited potent anti-HIV-1 protease activity with low or no cytotoxicity. Some of the isolated metabolites demonstrated cytostatic activities, which suggest further investigation of their use in concert with anti-viral drugs where the cytostatic compound arrests cell growth while the antiviral drug inhibits viral replication. It was mostly the polyhydroxylated-isolated metabolites that showed strong cytotoxicity and high selectivity in the cancer cells used in this study. The high cytotoxicity of polyhydroxylated sterols from marine organisms is well reported and supported by data presented here and highlights the importance of this skeleton in developing potential lead compounds from marine sources. Taking into consideration the advantage of the chemical structure similarity of these compounds to the human sterols, polyhydroxysterols can be considered as potential lead compounds for the development of new, safe, and effective medicines. Further studies on this type of active compound scaffold using structure activity relationship (SAR) modeling, could lead to the production of new compounds with even better activities and drug-like properties.

The biological activities of the *L. arboreum* metabolites have not been extensively studied meaning these metabolites may yet demonstrate additional biological activities. The compounds isolated from *L. arboreum* have been isolated from terrestrial organisms as well but appear to demonstrate more varied functionalities when isolated from a marine environment. If further investigation strongly indicates clinical value, the supply of active materials will have to be addressed by synthesis rather than continued isolation from live organisms, which is why SAR studies are crucial.

## Abbreviations

XTT: (2,3-bis-(2-methoxy-4-nitro-5-sulfophenyl)-2H-tetrazolium-5-carboxanilide)
ART: Antiretroviral therapy
HAART: highly active antiretroviral therapy
SI: Selectivity index
RT-CES: Real time cell electronic sensing
CI: cell index
AP: acetyl pepstatin
EIMS: Electron ionized mass spectrometry
